# Structural Characterization and Antifungal Studies of Zinc-Doped Hydroxyapatite Coatings

**DOI:** 10.3390/molecules22040604

**Published:** 2017-04-09

**Authors:** Simona Liliana Iconaru, Alina Mihaela Prodan, Nicolas Buton, Daniela Predoi

**Affiliations:** 1 National Institute of Materials Physics, Atomistilor Street, No. 105 bis, P.O. Box MG 07, 077125 Magurele, Romania; simonaiconaru@gmail.com; 2 Emergency Hospital Floreasca Bucharest, 8 Calea Floresca, Sector 1, 014461 Bucharest, Romania; prodan1084@gmail.com; 3 Surgery Department, Carol Davila University of Medicine and Pharmacy, 8 Eroii Sanitari, Sector 5, 050474 Bucharest, Romania; 4 HORIBA Jobin Yvon S.A.S., 6-18, rue du Canal, 91165 Longjumeau CEDEX, France; nicolas.buton@horiba.com

**Keywords:** zinc, hydroxyapatite, *C. albicans*, thin layer

## Abstract

The present study is focused on the synthesis, characterization and antifungal evaluation of zinc-doped hydroxyapatite (Zn:HAp) coatings. The Zn:HAp coatings were deposited on a pure Si (Zn:HAp_Si) and Ti (Zn:HAp_Ti) substrate by a sol-gel dip coating method using a zinc-doped hydroxyapatite nanogel. The nature of the crystal phase was determined by X-ray diffraction (XRD). The crystalline phase of the prepared Zn:HAp composite was assigned to hexagonal hydroxyapatite in the P6_3/m_ space group. The colloidal properties of the resulting Zn:HAp (x_Zn_ = 0.1) nanogel were analyzed by Dynamic Light Scattering (DLS) and zeta potential. Scanning Electron Microscopy (SEM) was used to investigate the morphology of the zinc-doped hydroxyapatite (Zn:HAp) nanogel composite and Zn:HAp coatings. The elements Ca, P, O and Zn were found in the Zn:HAp composite. According to the EDX results, the degree of Zn substitution in the structure of Zn:HAp composite was 1.67 wt%. Moreover, the antifungal activity of Zn:HAp_Si and Zn:HAp_Ti against *Candida albicans* (*C. albicans*) was evaluated. A decrease in the number of surviving cells was not observed under dark conditions, whereas under daylight and UV light illumination a major decrease in the number of surviving cells was observed.

## 1. Introduction

The development of modern medicine and bioengineering has created the premises for obtaining new and enhanced materials that could improve the quality of life of patients suffering from various diseases that lead to painful surgeries. One of the most alarming complication of orthopedic surgeries is the development of implant-associated infections [1]. These infections occur due to bacteria which adhere to the implant and colonize either the surface of the implant, or the tissue surrounding the surface of the implant [1,2,3]. These types of infections are known as implanted-cantered infections and are usually treated with very powerful antibiotics [4]. However, in the last decades, the number of antibiotic-resistant bacteria has increased, rendering systemic antibiotics inefficient [4,5]. As a result of these infections, the implants may loosen requiring revision procedures which are dangerous and very uncomfortable for the patients. According to recent studies [6,7,8,9], the number of surgeries necessary due to mechanical loosening of total hip replacements increases every year [8,9]. In this context, researchers and medics alike have come to the conclusion that a method for improving implants would be to coat them with a biomaterial enhanced with different antimicrobial agents [4,10] able to promote bone growth onto the implanted surface [8,11]. For this purpose, one of the most adequate biomaterial is synthetic hydroxyapatite (HAp). Synthetic hydroxyapatite (Ca_10_(PO_4_)_6_(OH)_2_) is very similar chemically and structurally to the natural mineral bone tissue [8,12]. It is used as a coating material for metallic implants due to its remarkable biocompatibility and osteoconductivity [8,13]. Earlier studies have shown that physicochemical bonds are formed between HAp and the surrounding bone tissue allowing new natural bone tissue to form around the implant thus achieving a good implant osseointegration [13,14]. In addition, a solution for enhancing the antimicrobial properties of HAp would be the integration into the hydroxyapatite structure of trace elements, such as Zn, Ag, Au, Cu, Ti or Cu [15,16]. One of the most widespread trace element involved in various functions of the human body is zinc which influences up to 200 enzymes [13,17]. Considering that 28% of the entire amount of zinc present in the body is found in the bone tissue and that its presence allows a better osteoblast proliferation as well as an enhanced biomineralization and bone formation [15,18,19,20], the substitution of some Ca ions by Zn ions could enhance the properties of HAp. Beside the role of zinc in maintaining membrane structure, protein synthesis and cell proliferation, it has been proven that zinc is also effective in inhibiting the growth of *C. albicans* [21,22]. Therefore, besides silver [23,24], zinc may play an important role in the creation of new antimicrobial agents. As a results, zinc-doped hydroxyapatite could be a solution for obtaining an improved biomaterial that could be used for various biomedical applications [25,26,27]. 

For this research Ti and Si substrates with Zn:HAp coatings (Zn:HAp_Ti and Zn:HAp_Si) with potential applications in the medical field were fabricated by a sol-gel dip coating method. In the following study, a detailed physico-chemical characterization of the Zn:HAp nanogel and Zn:HAp thin layers is provided. In addtion, the antifungal activity of the obtained Zn:HAp coating exposed to daylight, UV light and kept in the dark was studied in order to evaluate the role of the zinc from the hydroxyapatite structure in the fight against *C. albicans*.

## 2. Results and Discussion

**Figure 1:**
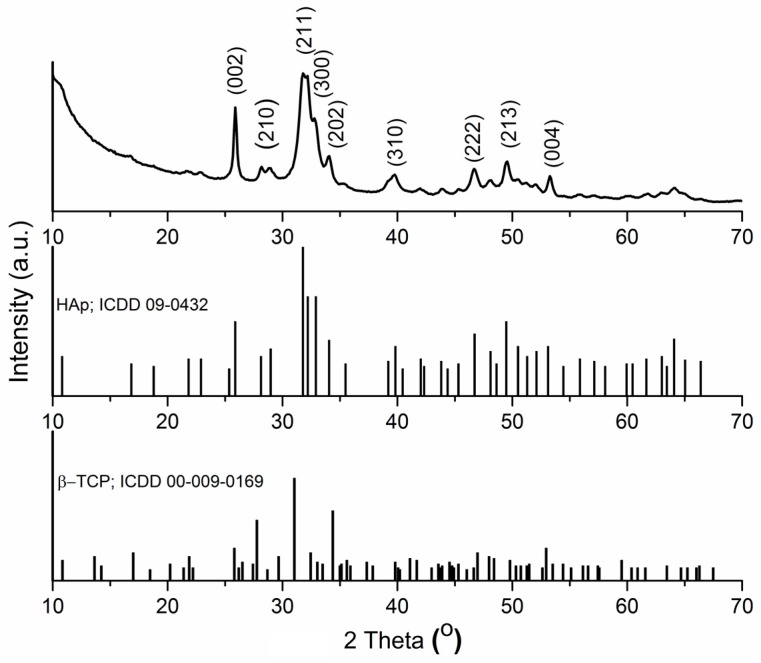
The XRD patterns of Zn:HAp powder obtaining by sol-gel method.

The XRD pattern of Zn:HAp powder (x_Zn_ = 0.1) prepared by sol-gel method and dried at 100 °C for 60 min is shown in Figure 1. The XRD spectrum of Zn:HAp powder presents peaks attributed to pure hexagonal HAp.

All the peaks present in the XRD spectrum of Zn:HAp powder are consistent with the Powder Diffraction File (PDF) standard card ICDD 09–0432. The XRD spectrum reveals sharp and strong peaks indicating a high degree of crystallinity of the Zn:HAp powder after thermal treatment at 500 °C. The typical peaks, (002), (210), (211), (300), (202), (310), (222), (213) and (004) confirm the presence of pure HAp. It can be observed that the peaks of rhombohedral β-tricalcium phosphate (β-Ca_3_(PO_4_)_2_, β-TCP) do not appear in the Zn:HAp sample according to ICDD card PDF file number 00-009-0169. Furthermore, no other peaks characteristic to any impurities were observed, suggesting a good purity of the Zn:HAp powder.

The present research confirmed previous studies reported by LeGeros et al. [28] and Miyaji et al. [29]. According to these studies, the substitution of Ca ions by Zn ions in the hydroxyapatite structure is possible because the ionic radius of Ca^2+^ ions (100 pm) is greater than that of Zn^2+^ (74 pm). Furthermore, in their recent studies on in vitro cytocompatibility and corrosion resistance of zinc-doped hydroxyapatite coatings on a titanium substrate, Ding et al. [30] have shown that the apatite structure has excellent flexibility in allowing substitutions of Ca^2+^ by Zn^2+^.

**Figure 2:**
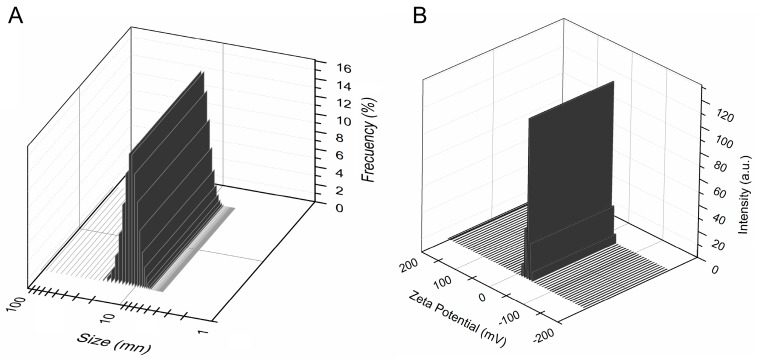
Particle size distribution measured by DLS (**A**) and Zeta potential (**B**) of Zn:HAp nanogel coating solution.

The colloidal feature of the resulted Zn:HAp (x_Zn_ = 0.1) nanogel solutions were examined by zeta potential and Dynamic Light Scattering (DLS). These characteristics are presented in Figure 2A,B. The zeta potential and particle sizes of Zn:HAp were determined from sol-gel coating solution after 30 min of ultrasonic agitation. Zeta potential is a measure of colloidal stability.

The zeta potential measurements indicate that the Zn:HAp nanogel coating solution had a negative charge. Moreover, the zeta potential value of the Zn:HAp nanogel coating solution was −28.88 mV (Figure 2A). For measuring particle size, we chose DLS, one of the most widely used techniques. Figure 2B shows the Zn:HAp sol size distribution measured by DLS at 25 °C. DLS studies have indicated that a single population is present in the Zn:HAp nanogel prepared solutions. The particle size distribution was centered at around 27 nm.

**Figure 3:**
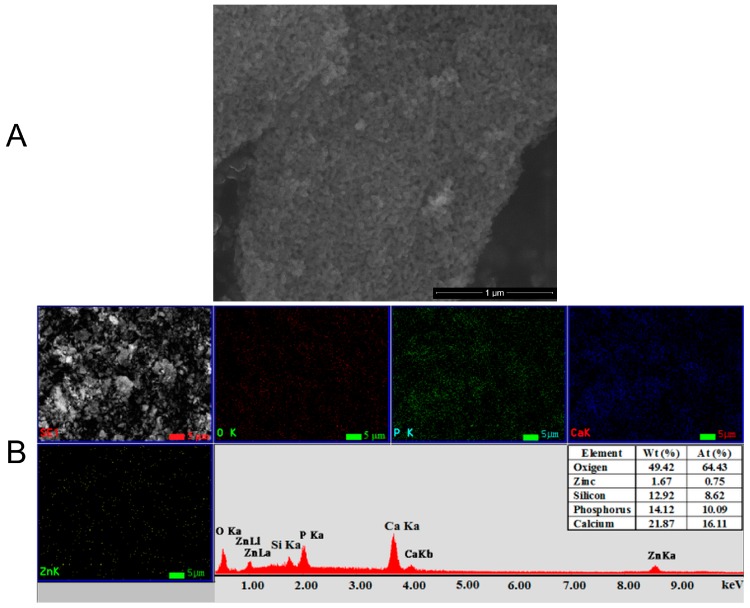
The SEM image (**A**), and elemental mapping, EDX spectrum and EDX analysis results (**B**) of Zn:HAp powder (x_Zn_ = 0.1) prepared by sol-gel method and dried at 100 °C for 60 min.

The morphology and chemical composition of Zn:HAp (x_Zn_ = 0.1) powder prepared by the sol-gel method and dried at 100 °C for 60 min were determined by SEM and EDX studies (Figure 3). In the SEM image (Figure 3A), the particles have dimensions between 10–50 nm and an average size around 25 nm and form agglomerates. In the EDX spectrum (Figure 3B) the peaks attributed to the chemical elements (Ca, P, O and Zn), characteristic to Zn:HAp powders could be observed. The homogeneous and uniform distribution of Ca, P, O and Zn in the sample was confirmed by the elemental mapping (Figure 3B). Besides, the results of EDX analysis of powder samples are also presented in Table inserted in the Figure 3B. On the other hand, ICP-MS studies were performed to investigate the quantity of zinc in the Zn:HAp (x_Zn_ = 0.1) powders. The amount of zinc in the ZnHAp powder calculated from the ICP-MS measurements was 14.36 mg/g. The result is in good agreement with previous studies by Predoi et al. [25].

**Figure 4:**
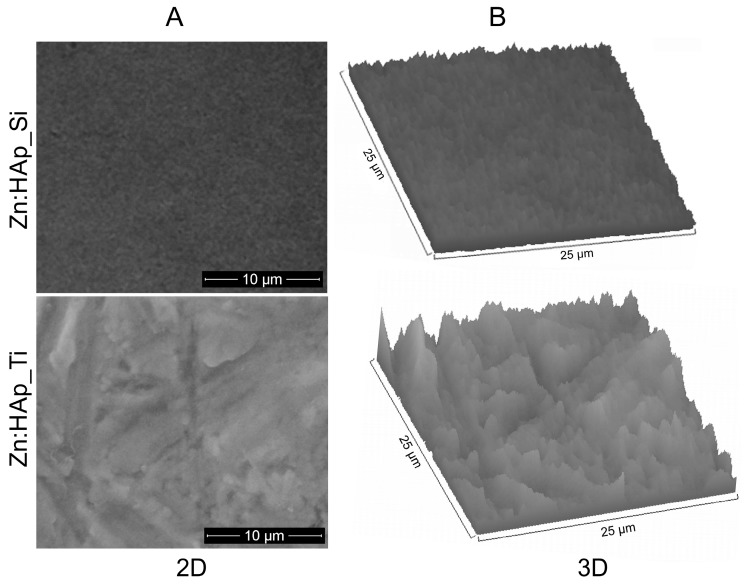
Surface micrographs (**A**) and 3D surface plots (**B**) of Zn:HAp_Si and Zn:HAp_Ti coatings.

Figure 4 illustrates the surface micrographs (Figure 4A) and 3D surface plots (Figure 4B) of Zn:HAp_Si and Zn:HAp_Ti coatings dried for 60 min at 100 °C and thermally treated in air for 60 min at 300 °C.

The texture of Zn:HAp coatings deposited on pure Si and Ti substrate was obtained by performing a 3D surface plot of their SEM images using Image J software. The surface morphologies of the Zn:HAp coatings deposited on a pure Si and Ti substrate were compared by SEM analysis. A difference in the morphology of the Zn:HAp_Si and Zn:HAp_Ti coatings can be noted, although a dense layer with a homogeneous structure was obtained in both cases. Zn:HAp layer covering the pure Si substrate is uniform while the Zn:HAp layer covering the Ti substrate shows a rougher surface. Bright regions corresponding to the surface of Si or Ti were not observed.

**Figure 5:**
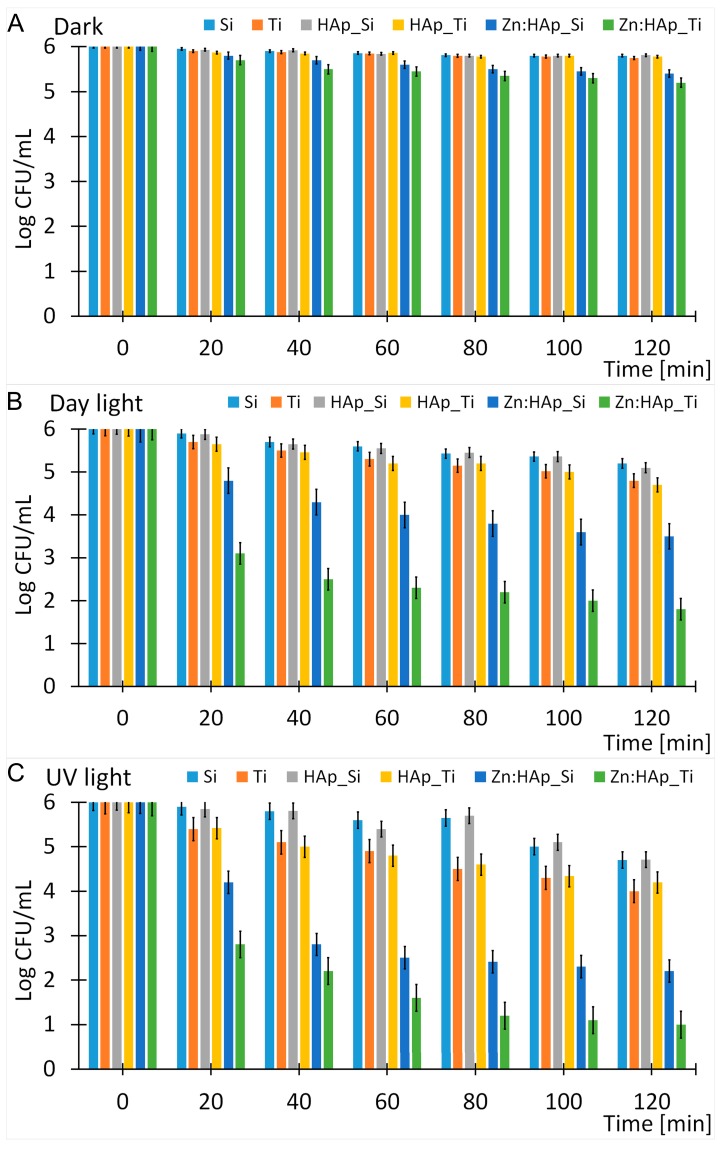
Survival curves of *C. albicans* on Ti, HAp_Ti, Zn:HAp_Ti, Si, HAp_Si, and Zn:HAp_Si substrate in the dark (**A**), exposed to day light (**B**) and UV light (**C**).

It is well known as the distribution widespread of infectious diseases, due to the emergence and development continue of antifungal treatments resistant yeasts, represents a big challenge for human health worldwide. According to Bliss et al. [31] *C. albicans* is the most important human fungal pathogen, causing serious illness that can provoke death while effective treatment is increasingly difficult [32]. Consequence of the complicated diagnosis of fungal infections, the mortality and morbidity caused by *C. albicans* are yet unacceptably high despite the existing antifungal therapies. Due to these major problems in treating fungal infections, researchers worldwide are trying to identify novel alternative treatments based on the development of new materials. The antifungal activity of new Zn:HAp coatings on substrates of Ti and Si against *C. albicans* was evaluated in different conditions (Figure 5). Moreover, the antifungal activity of HAp coatings on substrates of Ti and Si against *C. albicans* was evaluated in different conditions as reference. The development of *C. albicans* cultures grown under the same conditions kept in the dark and exposed to UV light and daylight were evaluated. These studies were used for a better understanding of the influence of both zinc ions and illumination on the eradication of *C. albicans* cells. The results presented in this study showed that the *C. albicans* cell survival was affected after exposure to UV light and daylight. We found that the survival ratio of *C. albicans* on the Zn:HAp coating kept in the dark decreased to an insignificant level after 120 min (Figure 5A). A negligible level regarding the survival ratio of *C. albicans* was also observed on the Ti and Si substrate after 120 min (Figure 5A). The survival ratio of *C. albicans* observed on the HAp_Ti and HAp_Si films at various time intervals and kept in the dark was not different from that of the Ti and Si substrate. According to these observations we can say that the HAp films did not show antifungal activity under these circumstances (Figure 5A). This comportment indicated that in the dark conditions the development of *C. albicans* cells was not affected. The slight decrease in the survival ratio of *C. albicans* cells observed when Si and Ti substrates were coated with Zn:Hap was attributed to zinc ions from the Zn:HAp composite layers (Figure 5A). The viability of *C. albicans* cells after exposure to UV light and daylight was dependent on the exposure time and the type of coating (Figure 5B,C). A negligible toxicity was detected for the *C. albicans* cells during the first 40 min after exposure to UV light (Figure 5B) and daylight (Figure 5C) of the Ti and Si substrate. After 120 min of exposure to daylight (Figure 5B) the *C. albicans* cell survival decreases by 1.2 and 0.8 log, respectively, in the case of the Ti and Si substrate. A decrease of *C. albicans* cell survival by 2 and 1.26 log was observed in case of Ti and Si substrate after exposure to UV light (Figure 5C). The survival ratio of *C. albicans* observed for HAp_Ti and HAp_Si films was insignificantly affected, relative to the survival ratio of *C. albicans* for Ti and Si substrate after exposure to UV light for the different intervals of time for which the evaluation was carried out (Figure 5B). However, a slightly increased toxicity for the *C. albicans* cells in presence of HAp_Ti films relative to the HAp_Si films (Figure 5B) was observed. A more effective action against *C. albicans* was observed when Ti and Si substrate with HAp coating and was exposed to UV light (Figure 5C). The behavior of HAp_Ti films relative to the HAp_Si films was the same as in samples exposed to daylight. The survival of *C. albicans* cells on Zn:HAp_Ti and Zn:HAp_Si films after 120 min exposure to UV light was only 1 log and 2.2 log, respectively (Figure 5C). A much less efficient action can be observed when Ti and Si substrate with Zn:HAp coating was exposed to daylight (Figure 5B). It was found that the survival of *C. albicans* cells after exposure to daylight for 120 min was 1.8 log (Zn:HAp_Ti) and 3.5 log (Zn:HAp_Si). It is apparent that the Ti substrate with Zn:HAp coating was the most efficient against *C. albicans* regardless of the illumination used.

**Figure 6:**
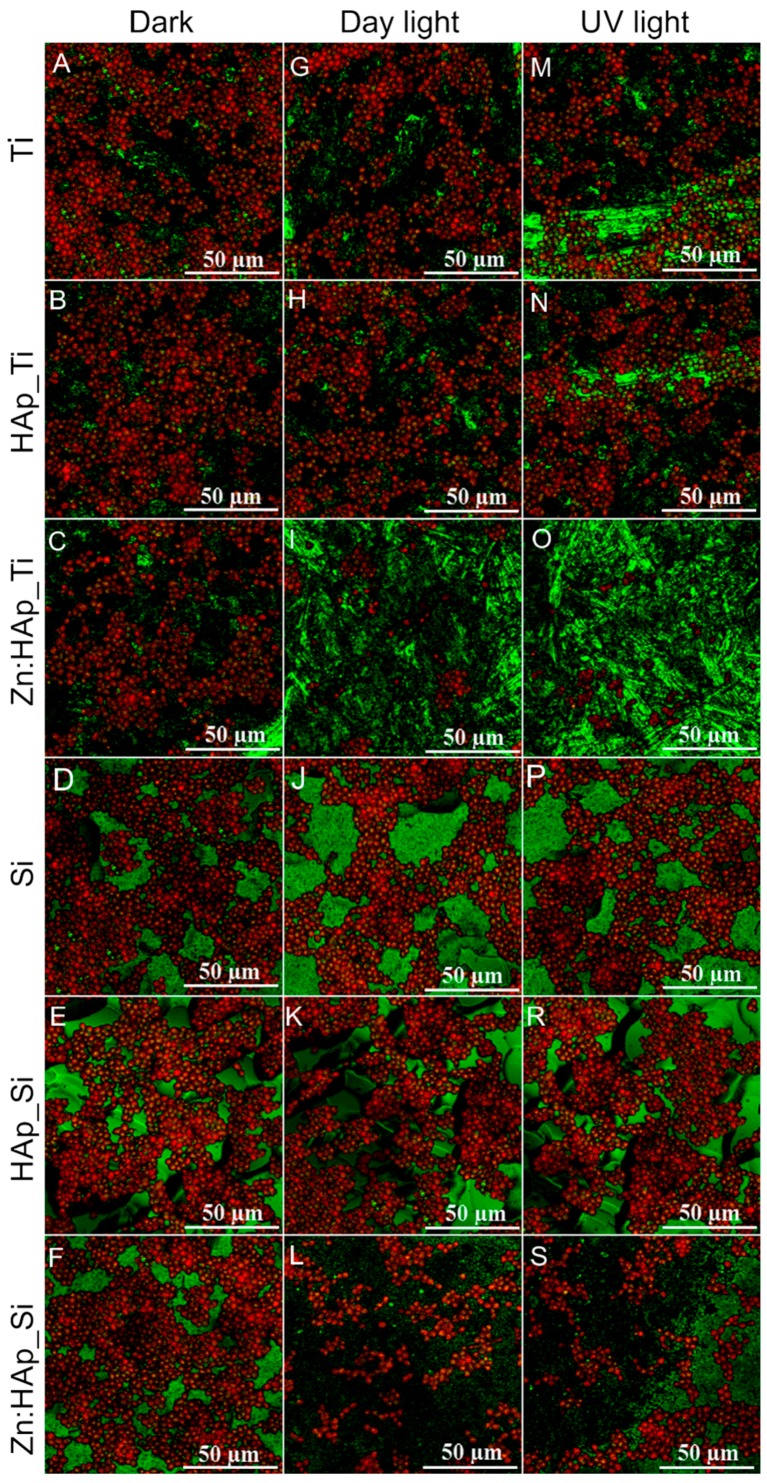
CLSM image of *C. albicans* adhesion on Ti (**A**, **G**, **M**), HAp_Ti (**B**, **H**, **N**) Zn:HAp_Ti (**C**, **I**, **O**), Si (**D**, **J**, **P**), HAp_Si (**E**, **K**, **R**), and Zn:HAp_Si (**F**, **L**, **S**) after exposure to day light, UV light and kept in the dark. Surviving cells appear red.

The adhesion of *C. albicans* cell on different surfaces (Ti, HAp_Ti, Zn:HAp_Ti, Si, HAp_Si, and Zn:HAp_Si) after exposure to daylight and UV light and when kept in the dark at 37 °C was analyzed by CLMS (Figure 6). A large number of surviving cells on Ti and Si substrate, on HAp_Ti, HAp_Si layers and on the Zn:HAp_Ti and Zn:HAp_Si layers were observed (Figure 6A–F). On the other hand, when the HAp_Ti, HAp_Si layers were exposed to daylight or UV light a slight influence was observed in reducing the number of surviving *C. albicans* cells. Nevertheless, in none of these cases can we speak of an antifungal effect, even though after exposure to daylight or UV light the cellular proliferation was lower. Furthermore, it can be seen that the antifungal behavior of HAp_Ti (Figure 6B,H,N) and HAp_Si (Figure 6E,K,R) layers is similar to that of the Ti and Si substrates (Figure 6A,E,I,D,J,P). Though, the number of surviving cells on HAp_Ti, HAp_Si layers decreased after exposure to daylight or UV light, this effect was not noticed in terms of CFU/mL (Figure 5). This behavior might suggest that HAp_Ti and HAp_Si layers had a temporary and reversible effect against the proliferation of *C. albicans* cells. The number of surviving cells on Ti and Si substrate and on the Zn:HAp_Ti and Zn:HAp_Si layers decreased when the samples were exposed to daylight (Figure 6G–L) and UV light (Figure 6M–S). In comparison, fewer surviving cells of *C. albicans* were found on Zn:HAp_Ti (Figure 6I,O) and Zn:HAp_Si (Figure 6L,S) samples. On the other hand, for Zn:HAp_Ti and Zn:HAp_Si samples, it can be seen that the number of surviving cells decreased when the samples were exposed to UV light (Figure 6O,S).

**Figure 7:**
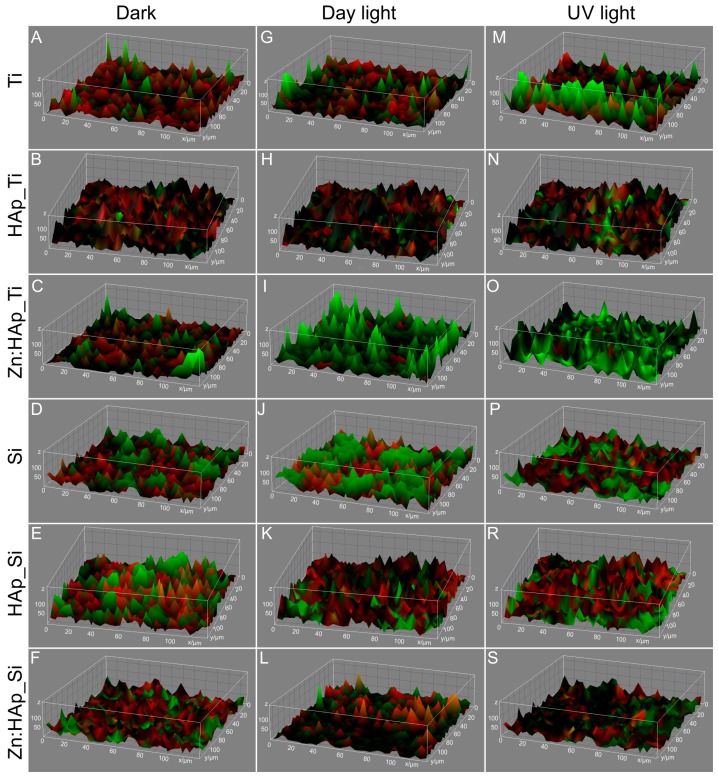
The 3D composite images of survival of *C. albicans* cells on Ti (**A**, **G**, **M**), HAp_Ti (**B**, **H**, **N**) Zn:HAp_Ti (**C**, **I**, **O**), Si (**D**, **J**, **P**), HAp_Si(**E**, **K**, **R**), and Zn:HAp_Si (**F**, **L**, **S**) after exposure to day light, UV light and kept in the dark. Surviving cells appear red.

The 3D composite images analyzed using Image J software [33] presented in Figure 7 displays the structure and spatial distribution of *C. albicans* surviving cells on different surfaces (HAp_Ti, Zn:HAp_Ti, HAp_Si, Zn:HAp_Si, Ti and Si) kept in the dark and after exposure to day light and UV light. The images exhibited in Figure 7 reveal the spatial distribution of *C. albicans* surviving cells (red color) along horizontal (coverage) and the vertical (thickness) distributions on Ti, PDMS, HAp-PDMS, Zn:HAp-PDMS and Ag:HAp-PDMS surfaces. The CLSM images presented in Figure 7 demonstrated that the survival of *C. albicans* cells have been significantly reduced in the presence of Zn:HAp_Ti layers (Figure 7I,O) depending on the light. A decrease of *C. albicans* surviving cells depending on the light exposure was also observed in the presence of Zn:HAp_Si layers (Figure 7L,S).

The potential use of layers, composed of Zn doped hydroxyapatite nanoparticles, in biological applications, by evaluating the antifungal activity against microbial cells (*C. albicans*) was studied in this research. The structure of Zn:HAp nanogel investigated by XRD was characteristic of pure HAp with a good purity of the Zn:HAp powder resulting from nanogel due to flexibility of substitution of Ca^2+^ by Zn^2+^. The antifungal activity of Ti and Si with Zn:HAp coating with a homogeneous structure evaluated after exposure to daylight, UV light and kept in the dark was affected by the exposure time. In addition, it was demonstrated that the antifungal activity of the Zn:HAp_Ti and Zn:HAp-Si layers after exposure to UV light was more efficient against *C. albicans*. Moreover, the antifungal activity of the Zn:HAp_Ti and Zn:HAp-Si layers was influenced by the surface morphology. The antifungal activity was not significantly influenced by the types of substrates according to Zajicova et al. [34]. According to our knowledge, this study is the only one investigating the effect of Zn:HAp coating on *C. albicans*. Certain studies have examined the effect of other thin layers on *C. albicans* [14]. Recent research regarding the effect of antimicrobial activity of Zn:HAp nanoparticles is available [25,26]. In recent studies on textural, structural and biological evaluation of hydroxyapatite doped with zinc at low concentrations, Predoi et al. [25] assessed the effect of Zn:HAp on cell viability on prokaryote and eukaryote cells. They showed that a significant cytotoxicity was observed on *S. aureus* while a very low cytotoxicity was observed on *E. coli*. On the other hand, the cytotoxic effects were intense in the case of hepatic cells. Furthermore, the studies on structural and biological assessment of zinc doped hydroxyapatite nanoparticles conducted by Popa et al. [26] concluded that that “Zn doping at the tested concentrations did not induce a specific prokaryote or eukaryote toxicity in HAp compounds” and clearly showed that the activity of Zn:HAp nanoparticles against HepG2 cells was meaningfully dependent on the particle size. Another study on flower-shaped ZnO nanoparticles synthesized by a novel approach at near-room temperatures with antibacterial and antifungal properties conducted by Khan et al. [35] showed that the antimicrobial effects can be influenced by the shape of nanoparticles. Phaechamud et al. [36] in their studies on the effects of types and amounts of ZnO on the antimicrobial activity were evaluated using *S. aureus*, *E. coli* and *C. albicans* as standard microbes, established that at low concentrations the growth of *S. aureus* was stronger inhibited than *C. albicans* or *E. coli*. A similar behavior has been also established by Zhang et al. [37] in their studies on ZnO nanofluids as a potential antibacterial agent. They showed that the particle size and shape affected the antibacterial activity and the antibacterial activity increases when the particle size decreases. Padmavathy et al. [38] attributed the enhanced antimicrobial activity of smaller particles to the higher surface area to volume ratio leading to a larger number of active oxygen species released from ZnO surface to kill bacteria more effectively. On the other hand, Hwang et al. [39] in their studies on silver nanoparticles demonstrated that silver nanoparticles induce apoptotic cell death in *C. albicans* by increasing the concentration of hydroxyl radicals. We can conclude that indifferent of the mechanism of action, the Zn:HAp coatings could be effective against *C. albicans*. For a better understanding of the behavior of these surfaces based on Zn:HAp in contact with various microbes, the effectiveness of these layers on different *C. albicans* fungal strains will be furthere studied. Finally, it can be said that our study opens new opportunities in the biomedical field by providing potential promising antifungal coating layers especially for use in hospitals for surgical devices and different biomedical implants.

## 3. Materials and Methods

### 3.1. Thin Layer of Zinc-Doped Hydroxyapatite

Zinc-doped hydroxyapatite, Ca_10−*x*_Zn(PO_4_)_6_(OH)_2_, (Zn:HAp) thin layers were prepared according to the method presented by Kim et al. [23]. The composition ratio, [Ca + Zn]/P, in the Zn:HAp sol was adjusted to be equal to 1.67 according to [24]. In order to obtain the compound that was later deposited by sol-gel dip coating method on a pure Si (Zn:HAp_Si) and Ti (Zn:HAp_Ti) substrate, triethyl phosphite (TEP), P(C_2_H_5_O)_3_, 1M, and zinc nitrate (Zn(NO_3_)_2_·6H_2_O) were dissolved in ethanol. After adding distilled water (OH/P = 10), the solution was stirred vigorously for 24 h at 40 °C. After that, a NH_4_OH solution (5 vol. %) was added to the solution containing P and Zn. Simultaneously, in a different beaker, a stoichiometric amount of calcium nitrate (Ca(NO_3_)_2_·4H_2_O) was dissolved in ethanol and stirred for 24 h at 40 °C. The solution containing Ca was added drop by drop in the solution containing P and Zn. The mixture was stirred continuously for a week at 40 °C. The resulted sol-gel solution was used to prepare by sol-gel dip coating method the Zn:HAp_Si and Zn:HAp_Ti composite layers. Afterwards, the disks coated with a Zn:HAp thin layer were dried for 60 min at 100 °C and thermal treated in air for 60 min at 500 °C in order to obtain films with a crystalline structure and to remove the solvents. On the other hand, a part of the sol-gel obtained was dried for 60 min at 100 °C and assayed morphologically and structurally.

### 3.2. Structural and Morphologycal Characterizations

A D8 Advance diffractometer (Bruker, Karlsruhe, Germany) was used in order to obtain the XRD patterns in the 2θ range 10°–70°. The diffractometer was equipped with a nickel filtered Cu K_α_ (λ = 1.5418 Å) radiation and a high efficiency one-dimensional detector (Lynx Eye type) operated in integration mode. The morphology and elemental composition of the Zn:HAp powder and layers deposited on a pure Si and Ti substrate were investigated using a Quanta Inspect F scanning electron microscope equipped with an energy dispersive spectroscope (EDX, MnK resolution at 133 eV). The colloidal properties of the resulted sol-gel Zn:HAp (x_Zn_ = 0.1) were analyzed by Dynamic Light Scattering (DLS) and zeta potential using dynamic light scattering (SZ-100 Nanoparticle Analyzer (HORIBA Ltd., Kyoto, Japan) at 25 ± 1 °C. All the samples were diluted in ethanol before analysis.

### 3.3. Laser Ablation-Inductively Coupled Plasma-Mass Spectrometer (LA-ICP-MS) Analysis

The samples were subjected to laser ablation-inductively coupled plasma-mass spectrometer (LA-ICP-MS) tests according to the procedure described by Motelica-Heino and Donard [40]. An elemental XR Thermo Specific instrument (Waltham, MA, USA), was used in combination with a UV laser probe laser ablation sampling device (Teledyne CETAC Technologies, Omaha, NE, USA). The Zn:HAp powders were prepared as small pellets for analysis. The repetition rate of the 266 nm wavelength laser was fixed to 10 Hz. Calibration was executed with certified artificial glass, NIST-610. Measurements were reproduced four times to verify the analytical accuracy of the technique.

### 3.4. In Vitro Antifungal Activity

Standard inoculum was achieved by passing a single colony of *Candida albicans* in nutrient broth and incubated at 37 °C for 12 h. A 5 McFarland standard (10^6^ CFU/ml) for *C. albicans* ATCC 10231 was prepared. The Zn:HAp and HAp thin layers deposited on a pure Si and Ti substrate and also Si and Ti substrate were covered with 300 μL of fungal inoculum at a concentration of 10^6^ CFU/mL^−1^ in a Petri dish. The plates with Zn:HAp_Ti, Zn:HAp_Si, HAp_Ti, HAp_Si, pure Si and Ti were incubated in the dark at 37 °C for 30 min without being stirred. One hundred μL of the suspensions from the original plate was transferred onto a new plate and exposed to daylight and to UV light intensities of 1 μW/cm^2^. On the other hand, a 100 μL suspension of the original plate was used as a control and kept for the time of irradiation in the dark at 37 °C. Every 20 min during 120 min, 10 μL of the bacterial suspension were re-suspended in Yeast Nitrogen Base Broth (YNB, Difco, Detroit, MI, USA) enriched with 100 mM glucose. After that, the agar plates were incubated for 24 h at 37 °C and the final number of viable cells (CFU) was evaluated.

The survival of *C. albicans* cells on different surfaces (Ti, HAp_Ti, Zn:HAp_Ti, Si, HAp_Si, and Zn:HAp_Si) of composite layers was investigated using confocal laser scanning microcopy (CLSM). For the CLSM observation, the cells were stained for 5 min with 1 mg/mL ethidium bromide (EtBr) in dark, washed two times with water, air dried and then visualized in reflection and fluorescence modes by using a SP confocal microscope (TCS Leica, Wetzlar, Germany) equipped with a 10× HCX PL FLUORITE objective, with a numerical aperture NA of 0.3. In order to acquire both reflection and fluorescence, an Ar ion laser (488 nm) was used.

## 4. Conclusions

In this research, the sol–gel method was used to prepare Zn:HAp powders. The Ti and Si surfaces with Zn:HAp and HAp coatings were obtained by a sol-gel dip coating method. The nanogel obtained was studied morphologically and structurally by XRD and SEM. The presence of the constituent elements of Zn:HAp were evaluated by EDX analysis. The colloidal characteristics of the resulted Zn:HAp nanogel solutions were also evaluated by zeta potential and DLS. In addition, the morphology of the Zn:HAp_Ti and Zn:HAp-Si coatings was investigated by SEM. The Zn:HAp_Ti and Zn:HAp_Si coatings were tested against a *C. albicans* microbial strain under exposure to daylight, UV light and when kept in the dark. CLSM was used to investigate the spatial distribution of *C. albicans* surviving cells on different surfaces kept in the dark and after exposure to daylight and UV light. It was demonstrated that the surviving cells were no longer dominant when the Zn:HAp_Ti and Zn:HAp_Si samples were exposed to UV light. The excellent antifungal activity of the prepared coatings create premises that might be applied on different types of surfaces which could be used in the future for various applications in biomedical fields.
